# Challenges in Identifying and Determining the Impacts of Infection with Pestiviruses on the Herd Health of Free Ranging Cervid Populations

**DOI:** 10.3389/fmicb.2016.00921

**Published:** 2016-06-17

**Authors:** Julia F. Ridpath, John D. Neill

**Affiliations:** Ruminant Diseases and Immunology Research Unit, National Animal Disease Center, Agricultural Research Service, USDAAmes, Iowa

**Keywords:** pestivirus, cervids, wildlife diseases, surveillance, sampling

## Abstract

Although most commonly associated with the infection of domestic livestock, the replication of pestiviruses, in particular the two species of bovine viral diarrhea virus (BVDV), occurs in a wide range of free ranging cervids including white-tailed deer, mule deer, fallow deer, elk, red deer, roe deer, eland and mousedeer. While virus isolation and serologic analyses indicate that pestiviruses are circulating in these populations, little is known regarding their impact. The lack of regular surveillance programs, challenges in sampling wild populations, and scarcity of tests and vaccines compound the difficulties in detecting and controlling pestivirus infections in wild cervids. Improved detection rests upon the development and validation of tests specific for use with cervid samples and development and validation of tests that reliably detect emerging pestiviruses. Estimation of impact of pestivirus infections on herd health will require the integration of several disciplines including epidemiology, cervid natural history, veterinary medicine, pathology and microbiology.

## Introduction

The recognized species of the Pestivirus genus include bovine viral diarrhea virus types 1 (BVDV1) and 2 (BVDV2), classical swine fever virus (CSFV), and border disease virus (BDV) (Simmonds et al., 2012). In addition to these four species, five putative species have been proposed; Bungowannah virus, giraffe virus, HoBi-like virus, pronghorn virus (PHV) and atypical porcine pestivirus. All four of the recognized species have been isolated from free ranging wildlife populations and two of the putative species, giraffe virus and PHV, have only been isolated from free ranging wildlife species (Table 1). Despite abundant evidence that pestiviruses currently circulate in wildlife populations, the full impact of exposure and prevalence of these infections are largely unknown. The limited information available regarding prevalence is mainly in the form of serological surveys (Table 2). Even though these studies have been limited and sporadic, they have demonstrated that a wide range of wildlife species havea wide range of wildlife species has been infected by pestiviruses. Further, controlled studies have shown that pestiviruses infect wild species and once infected they may transmit virus (Grondahl et al., 2003; Uttenthal et al., 2005, 2006; Duncan et al., 2008a; Nelson et al., 2008; Passler et al., 2010; USDA, 2010; Pruvot et al., 2014). While it is possible that positive serology results may be due to contact with domestic species, the high prevalence of seropositive samples within some isolated wild life populations without close contact with domestic species suggest that pestiviruses are being maintained independently within wildlife populations. This is illustrated by a study in which the geographic location of BVDV antigen-positive cattle and BVDV-seropositive white-tailed deer were analyzed using the dual kernel density estimation method. An exploratory cluster analysis revealed 1 significant cluster of BVDV antigen-positive herds and 2 significant clusters of BVDV-seropositive deer. There was no spatial overlap between the clusters suggesting that BVDV is maintained independently in domestic livestock herds and in the white-tailed deer population.(Kirchgessner et al., 2013).

**Table 1:** **Detection of pestivirus species in samples collected from free ranging wildlife populations**.

**Pestivirus species**	**Family**	**Wildlife Population**	**Country**	**References**
BVDV1 and BVDV2	Cervidae	White-tailed deer (*Odocoileus virginianus*)	US	Chase et al., 2008; Passler et al., 2008
BVDV1 BVDV2		Mule deer (*Odocoileus hemionus*)	US	Van Campen et al., 2001; Duncan et al., 2008b; Wolff et al., 2016
BVDV		Scottish red deer (*Cervus elaphus scoticus*)	Scotland	Nettleton et al., 1980
BVDV1		European roe deer (*Capreolus capreolus*)	Germany	Fischer et al., 1998
BVDV		Water deer (*Hydropotes inermis*)	South Korea	Kim et al., 2014
BVDV1		Sika deer (*Cervus nippon*)	China	Gao et al., 2011
BVDV1	Bovidae	Bighorn sheep (*Ovis canadensis*)	US	Wolff et al., 2016
BVDV1		Mountain goat (*Oreamnos americanus*)	US	Wolff et al., 2016
BDV		Pyrenean chamois (*Rupicapra pyrenaica pyrenaica*)	Pyrenees (border between France and Spain)	Arnal et al., 2004
BVDV1		Canadian bison (*Bison bison bison*)	Canada	Deregt et al., 2005
CSFV	Suidae	Wild boar (*Sus scrofa*)	France	Simon et al., 2013
Giraffe	Giraffidae	Giraffe (*Giraffa camelopardalis*)	Kenya	Plowright, 1969
Pronghorn	Antilocapridae	Pronghorn (*Antilocapra americana*)	US	Vilcek et al., 2005

**Table 2:** **Free ranging species with reported titers against pestiviruses**.

**Family**	**Species**	**Geographic regions**	**References**
Bovidae	Gemsbok (*Oryx gazella*), Roan antelope (*Hippotragus equinus*), Blue wildebeest (*Connachaetes taurinus*), Kudu (*Tragelaphus strepsiceros*), Eland (*Taurotragus oryx*), Buffalo (*Syncerus caffer*), Nyala (*Tragelaphus angasi*), Waterbuck (*Kobus ellipsiprymnus*), Defrassa waterbuck (*Kobus defrassa*), Lechwe (*Kobus leche*), Reedbuck (*Redunca arumdinum*), Sable antelope (*Hippotragus niger*), Oryx (*Oryx gazella*), Tsessebe (*Damaliscus lunatus*) Hartebeeste (*Alcelaphus buselaphus*), Wildebeeste (*Connachaetes taurinus*), Impala (*Aepyceros melampus*), Springbok (*Antidorcas marsupialis*), Duiker (*Sylvicapra grimmia*), Chamois (*Rupicapra pyrenaica pyrenaica*), Mouflon (*Ovis orientalis*), Bighorn sheep (*Ovis canadensis*), European bison, American bison	Africa, North America, Europe	Hamblin and Hedger, 1979; Depner et al., 1991; Marco et al., 2011; Wolff et al., 2016
Cervidae	Water deer (*Hydropotes inermis*), Reindeer/Caribou (*Rangifer tarandus*), Roe deer (Capreolus capreolus), Red deer (Cervus elaphus), Moose (Alces alces) Fallow deer (*Dama dama*), white-tailed deer (*Odocoileus virginianus*), mule deer (*Odocoileus hemionus*), Sika deer (*Cervus nippon*)	Asia, North America, Europe	McMartin et al., 1977; Lawman et al., 1978; ElAzhary et al., 1979; Couvillion et al., 1980; Van Campen et al., 2001; Lillehaug et al., 2003; Kim et al., 2014; Wolff et al., 2016
Giraffidae	Giraffe (*Giraffa camelopardalis*)	Africa	Hamblin and Hedger, 1979; Depner et al., 1991
Antilocapridae	Pronghorn antelope (*Antilocapra americana*)	North America	Barrett and Chalmers, 1975
Camelidae	Vicuna (*Vicugna vicugna*)	South America	Marcoppido et al., 2010
Suidae	Wild boar (*Sus scrofa*), Wart hog (*Phacochoerus aethoiopicus*)	Europe, Africa	Hamblin and Hedger, 1979
Leporidae	European rabbit (*Oryctolagus cuniculus*)	Europe	Frolich and Streich, 1998

The purpose of this article is to review reports regarding pestivirus infections in wild cervids and to summarize some of the challenges involved in determining the impact of pathogens infecting free ranging cervids.

## Surveillance based on detection or isolation of pestiviruses

Pestiviruses, principally BVDV1 and BVDV2, have been detected in samples collected from free ranging cervid populations (Table 1). However, isolations or detection by PCR tend to be a rare event among the populations surveyed (for references see Table 1). Cattle may be acutely or persistently infected with BVDV (Evermann and Barrington, 2005). Similarly it has been demonstrated that, under experimental conditions, cervids may be acutely or persistently infected with pestiviruses such as BVDV1 or BVDV2 (Passler et al., 2007, 2009; Ridpath et al., 2007, 2008). Experimental infections with typical field strains of BVDV in immunocompetent cattle and white tailed deer tend to be mild or asymptomatic (Ridpath et al., 2007, 2013). The majority of the surveys conducted to date relied on serum or ear notch samples which, at least in cattle, are better for detecting persistent infections than acute infections (Ridpath et al., 2002; Liebler-Tenorio et al., 2004). Further, based on the pattern of viral antigen present in various tissues it appears that the pestivirus positive deer harvested from free ranging populations were probably persistently rather than acutely infected. In cattle, persistently infected animals make up less than one percent of the population at slaughter but have a significant impact on the health of cohorts (Hessman et al., 2009). The detection of persistently infected animals (PI) in any population, domestic or free ranging, is significant as PIs act as efficient vectors for keeping the virus in circulation. However, persistent infections in deer are only established if the fetus is infected in the first one third of pregnancy (Ridpath et al., 2008, 2012). Thus, infections of the fetus occurring during the final two thirds of pregnancy and all infections of animals post-birth result in acute infections rather than persistent infections. Failure to detect acutely infected animals will lead to underestimation of infection rate. Therefore, while detection of PIs yields significant information it cannot be used as a measure of prevalence of infection.

## Serological surveys

Antibodies against pestiviruses have been identified from serum collected from seven different families of free ranging wildlife; Antilocapridae, Bovidae, Giraffidae, Cervidae, Suidae, Camelidae, and Leporidae with the greatest number of wildlife host species in the Bovidae and Cervidae families (Table 2). In North America, the largest numbers of wild ruminants are found in the Cervidae family (Flather et al., 2009) with five species being represented: moose (*Alces alces*), elk/wapiti (*Cervus elaphus*), caribou/reindeer (*Rangifer tarandus*), mule deer (*Odocoileus hemionus*), and white-tailed deer (*Odocoileus virginianus*) (Conner et al., 2008). Pestivirus neutralizing antibodies have been detected in free ranging populations of all five species (Table 2). A limitation of serological surveys is that the level of antigenic cross reactivity between pestivirus species makes it difficult to absolutely identify the pestivirus species elicited the immune response (Dubovi, 2013).

Many serological surveillance studies in wildlife arise out of pestivirus control programs aimed at clearing a pestivirus species, such as BVDV1 and BVDV2, from domestic animal populations. The primary goal of many of these studies is to determine if wildlife species can serve as virus reservoirs for domestic species, not to determine the level of infection in wildlife populations. The significant problem with these serological surveillance studies is that the level of neutralizing antibodies is only determined against one of the four recognized pestivirus species and this may result in underestimation of infection with emerging pestivirus species. This was noted by the authors in one of the earliest large scale serology surveys of wildlife which used samples collected from free ranging ungulates residing in Africa (Hamblin and Hedger, 1979). This survey evaluated 3359 sera, collected from multiple species of wildlife in nine African countries, for neutralizing antibodies against BVDV. At that point in history, the BVDV2 species had not yet been identified. Thus, the laboratory reference strains used in this study only belonged to the BVDV1 species. Neutralizing antibodies were detected in sera from 17 different species. The authors noted that because pestiviruses are cross reactive it is possible that the serum neutralizing antibodies reported in their study, may be due to cross neutralization with “other viruses as yet unrecognized.” It is also highly possible that antibodies against pestiviruses with limited cross reactivity with BVDV1 could have been missed in this and other studies.

Aside from an interest in pestiviruses that impact domestic species there are other reasons for the use of classic pestivirus strains in assays. Firstly, cytopathic reference strains from each of these species are readily available. This is not true of all emerging pestivirus species. To date only noncytopathic strains of the Giraffe and Pronghorn species are available. When cytopathic strains are used in virus neutralization (VN) tests, end points may be determined by observation of the cell monolayer. End point determination using noncytopathic strains requires secondary detection methods such as immunofluorescence, immunohistochemistry staining or polymerase chain reaction. Use of such secondary detection methods is time and cost prohibitive for large-scale surveillance projects.

Another consideration is that frequently emerging viruses, such as pronghorn virus (Vilcek et al., 2005) or atypical porcine pestivirus (Hause et al., 2015), do not initially grow well in cell lines commonly used in the laboratory (Vilcek et al., 2005). Finally, the pestivirus that the wild population was infected with may not yet have been isolated and characterized.

While there are valid reasons why serological surveys, based on VN tests, use reference strains from the four recognized species, it is highly probable that when these assays are used in such surveys they miss titers resulting from exposure to emerging viruses that are genetically distant and antigenically distinct. The greater the genetic difference between pestiviruses, the lower the cross reactivity (Ridpath et al., 2010; Bauermann et al., 2012). For example, the emerging bovine pestivirus species known as HoBi-like virus, while distinct, is closer to the two BVDV species than to other emerging pestivirus such as pronghorn virus. In one study it was shown that a serum collected from a bovid infected by a HoBi-like viruses had a greater than 1/500 titer against a HoBi-like virus, averaged a greater than 1/300 titer against BVDV2 strains but did not neutralize the pronghorn virus (Bauermann et al., 2012).

While commercial ELISA kits are available for detecting antibodies against the classic pestiviruses, particularly BVDV, the limited cross reactivity that exists between emerging pestiviruses and classic pestiviruses make these tests unreliable for detecting antibodies resulting from infection by emerging pestiviruses (Bauermann et al., 2012). Further, these commercial tests are not designed to differentiate between antibodies raised against different pestivirus species.

While performing serology on a one time collection of samples from a population can give information on the occurrence and prevalence of exposure, it does not yield information on when the exposure occurred. To estimate time of exposure, multiple samples over time must be collected and archived.

## Challenges in the collection of representative samples

Ideally samples should be representative of the population under study including biological, spatial, and temporal variables (Stallknecht, 2007). Further, samples must be collected while virus is present in tissues and tissues must be tested using technologies that maximize the probability of detecting the agent (Thurmond, 2003). Issues of access, cost and feasibility frequently preclude the gathering of such ideal samples.

If the goal is to detect a pestivirus the sample must be collected while the animal is still viremic. This not a problem with persistently infected cervids but is a problem with acutely infected cervids where the window of detectable viremia may be less than 5 days (Ridpath et al., 2007).

Both passive and active surveillance systems may be used to obtain cervid samples. Passive surveillance, which relies upon the observation and subsequent testing of an animal displaying clinical signs of disease or collection of samples from animals that have died of disease, is problematic for detecting infection with viruses, such as pestiviruses, which don't cause severe clinical disease. Passive surveillance tends to under estimate the impact of diseases that have significant mortality rates let alone those that result in subclinical disease. This is illustrated by an outbreak of hemorrhagic disease in white-tailed deer that occurred in Missouri. While it was estimated that the outbreak resulted in an 8% mortality rate, not one case of mortality or morbidity was reported by the public. The occurrence and extent of the outbreak were only noted because of surveillance conducted on 100 radio-monitored deer (Beringer et al., 2000). Some surveys for BVDV in deer have depended on getting samples from deer that were harvested by hunters (Duncan et al., 2008b; Passler et al., 2008). Hunting licenses usually require that the harvested animals are adults and most hunters desire to harvest healthy specimens. Thus, hunter harvested samples tend to represent healthy animals that have lived to sexual maturity, and based in studies in cattle, restricting surveys to healthy adults may result in underestimation of the incidence of persistent infection. In cattle it has been observed that animals persistently infected (PI) with BVDV are more frequently found among young stock than older stock because some (but not all) PI cattle succumb in the first year of life (Houe, 1992).

Even though hunter harvested samples may be skewed against including PI animals, BVDV PI animals have been detected in these samples (Van Campen et al., 2001; Chase et al., 2004; Duncan et al., 2008b; Passler et al., 2008) albeit at a low rate varying from 0.03 to 0.2%. The presence of PI deer indicates that BVDV circulates in these populations; however, their impact is difficult to assess.

The design of active surveillance systems requires an understanding of the social organization of the species to be studied. Unlike domestic livestock, wild deer do not confine their activities to large herd groups, cannot be rounded up without damaging ecosystems and social grouping, and are not amenable to handling. Populations are frequently divided into small breeding groups based on age and gender and contact between groups and make up within groups may change with the season. Neonates are frequently hidden rather than grazing with the herd.

The ideal surveillance program would include samples collected at multiple time points allowing retrospective analysis (Stallknecht, 2007). Archived samples are fundamental to estimating the introduction of a pathogen or detecting an increase in the incidence of infection.

## Assessing the impact of pestivirus infections

It is easier to assess the impact of infection with high virulence pestivirus strains that result in clinically severe acute disease such as classic swine fever in swine or hemorrhagic syndrome in cattle. However, the impact of lower virulence pestiviruses is harder to assess, even in domesticated species. Previous studies using captive deer have demonstrated white-tailed deer infected by pestiviruses such as BVDV1, BVDV2, and PHV display very mild clinical signs even though they are undergoing significant immune suppression (Van Campen et al., 1997; Vilcek et al., 2005; Ridpath et al., 2007, 2008, 2012). While the immune suppression may lead to reduction in herd health and numbers, the contribution of pestivirus infections to the problem may be difficult to establish. The prevalence of BVDV persistent infection in cattle, while low, has significant impact on production. Lonergan et al. determined that while PI cattle represent only 0.3% of the cattle population on arrival in feedlots, they accounted for 2.6% of chronically ill cattle and 2.5% of cattle that died during the observation period (Loneragan et al., 2005). Perhaps more importantly, exposure to PI animals has a significant impact on the health of cohorts. In the same study it was found that the risk of initial treatment for respiratory tract disease was 43% greater in cattle exposed to a PI animal, compared with those not exposed to a PI animal. Overall, 15.9% of initial respiratory tract disease events were attributable to exposure to a PI animal. In a subsequent study, Hessman et al. (2009) demonstrated that aside from overt disease, growth rates and feed conversion were negatively affected by the presence of PI cattle in feedlots. Comparing cattle lots with direct exposure to a PI with those without direct exposure revealed significant deficits in all performance outcomes associated with PI exposure. In the wild, where the rule is survival of the fittest, pestivirus infections which reduce efficiency in feed conversion and resistance to disease could be instrumental in a decline in animal numbers and population health.

## Conclusions

The limited serologic surveillance that has been published focused on the levels of neutralizing antibodies against the recognized pestivirus species. Such studies may underestimate exposure to emerging pestiviruses. The value of serological studies is greatly enhanced if sequential testing of the same population over is conducted. Samples, collected from the same population, over time allows detection of changes in exposure patterns.

Many studies rely on samples generated from deer harvested by hunters. However, such samples may yield skewed data as the majority of hunter-generated samples come from healthy, primarily male adults. Further, the tests available are designed for detection of recognized pestiviruses in domestic species. The reagents used may not be appropriate for wild cervids or emerging pestiviruses that are only distantly related to the recognized pestivirus species. In particular, cell cultures derived from domestic species may not work for the propagation of viruses that are adapted to cervid hosts. In summary, the full impact of pestiviruses on cervid populations may not be recognized at this time.

Improved detection rests upon the development and validation of tests specific for use with cervid samples and the development and validation of tests that reliably detect emerging pestiviruses. Estimation of the impact of pestivirus infections will require the integration of several disciplines including epidemiology, cervid sociology, veterinary medicine, pathology and microbiology.

## Author contributions

JR organized and drafted article. JN reviewed and amended article. Both authors agree to be accountable for this work.

### Conflict of interest statement

The authors declare that the research was conducted in the absence of any commercial or financial relationships that could be construed as a potential conflict of interest.
